# Children and young people at the intersection of chronic illness and migration: a scoping review

**DOI:** 10.1186/s44263-025-00131-3

**Published:** 2025-03-03

**Authors:** Brenda Agyeiwaa Poku, Lucy Hunt, Alison Pilnick, Karl Michael Atkin, Catrin Evans, Emily Pulsford, Susan Kirk

**Affiliations:** 1 School of Sociology and Social Policy, University of Nottingham, University Park, Nottingham, NG7 2RD UK; 2 School of Nursing and Public Health, Manchester Metropolitan University, Manchester, UK; 3 Department of Sociology, University of York, York, UK; 4 The Nottingham Centre for Evidence-Based Healthcare, School of Health Sciences, University of Nottingham, Nottingham, UK; 5 School of Medicine and Population Health, University of Sheffield, Sheffield, UK; 6 Division of Nursing, Midwifery and Social Work, School of Health Sciences, University of Manchester, Manchester, UK

**Keywords:** Children, Chronic illness, Intersectionality, Migration, Scoping review, Young people

## Abstract

**Background:**

Chronic illnesses (CIs) are increasingly prevalent among children/young people (CYP) globally. For migrant CYP with CIs, achieving a stable life in a new country can be particularly challenging due to additional barriers such as cultural and language differences, unsafe living conditions, and discrimination. While migration can sometimes improve healthcare access by introducing new models of care and ways of understanding health, these advantages are often outweighed by obstacles that hinder access to essential services. This review aimed to map the global evidence on post-migration experiences and outcomes of CYP with CIs and to identify priorities for research, policy, and practice to improve their care.

**Methods:**

A scoping review was conducted following JBI guidelines. We searched seven online databases, including MEDLINE, Embase, PsycINFO, Cochrane Library, CINAHL, Social Science Collection, and Web of Science, up to February 2024. Data were synthesised using a socio-ecological model, and four young migrants living with sickle cell disease in the UK contributed to the review through a Patient and Public Involvement Advisory Group.

**Results:**

Of the 58 included papers, most focused on migration to high-income countries, particularly the USA, and used institutional records or case studies. Few studies provided detailed information about migration status or reason for migration, often using proxies like parental country of birth or language spoken. The socio-ecological model revealed disparities in health status, treatment access, and health outcomes for migrant CYP with CIs. Key challenges were language, communication, costs, bureaucracy, family dynamics, coordination issues, resource constraints, and socio-political influences. Significant gaps included a lack of intersectional analyses (e.g. accounting for ‘race’ and citizenship) and limited qualitative research capturing the lived experiences of migrant CYP with CIs.

**Conclusions:**

Migrant CYP with CIs face significant health disparities shaped by individual, social, and systemic factors. Addressing these challenges requires intersectional and qualitative research, alongside collaboration with policymakers, practitioners, and communities, to inform more equitable healthcare policies and practices.

**Supplementary Information:**

The online version contains supplementary material available at 10.1186/s44263-025-00131-3.

## Background

Chronic illnesses, such as asthma, diabetes mellitus, sickle cell disease, thalassaemia, cystic fibrosis, cancer, epilepsy, and chronic kidney disease, are conditions that currently cannot be cured but can be managed through medication and therapies [1, 2]. These conditions, which often disrupt daily life and activities [3], are increasingly prevalent among children and young people (CYP) worldwide, with estimates ranging from 10 to 28% depending on the region [4]. Advances in medical care have improved survival rates and quality of life for CYP with chronic illnesses, with survival rates for childhood cancer now exceeding 80% in high-income countries like the USA [5]. These advancements have also led to better educational and employment outcomes, with 81% of CYP with chronic illnesses in the USA graduating high school and 60% entering the workforce [5]. However, managing chronic illness requires continuous engagement with healthcare systems, complex treatment regimens, regular medical monitoring, lifestyle adjustments, and additional care from parents and caregivers [6, 7]. These factors present unique physical and psychosocial challenges for CYP, influencing their development and well-being [8–12].

For CYP who have migrated internationally, managing chronic illness while adapting to a new country introduces additional layers of complexity. Migration, recognised as a key social determinant of health, significantly impacts morbidity and mortality, with host countries having a responsibility to ensure equitable healthcare access for migrants, as emphasised by the UN Sustainable Development Goals [13, 14]. Socioeconomic conditions in host countries play a substantial role in shaping migrant health outcomes, with barriers such as language differences, unfamiliar healthcare systems, and low health literacy limiting access to care [15–18]. Particularly vulnerable are undocumented migrant CYP, with 58% in Europe lacking healthcare access [19]. Migrant CYP also face risks related to migration itself, such as deteriorating health, acculturation stress, and trauma, especially for refugees and asylum seekers [17, 20–26]. Up to 30% of refugee and asylum-seeking CYP experience post-traumatic stress, depression, and/or anxiety [27].

For CYP with chronic illnesses, migration represents a disruption of their established routines, knowledge, and sense of self, requiring them to continuously adapt to new cultural, social, and healthcare environments. This transition can necessitate redefinitions of illness, patienthood, and self-management while also challenging them to navigate societal expectations from healthcare providers, educators, and employers [28, 29]. Migration-related barriers, including unsafe living conditions, fragmented services, language and cultural barriers, and uncertainty about healthcare entitlements, can further impede access to care and exacerbate health conditions [30, 31]. These challenges can lead to unmet health needs, disengagement from healthcare services, inadequate illness management, and long-term negative health outcomes, making migrant CYP with chronic illnesses particularly vulnerable.

However, some research highlights an ‘immigration paradox’, where migrant children sometimes fare better than their non-migrant peers despite facing socioeconomic challenges [32, 33]. Studies with adult migrants also suggest that positive coping strategies and adaptation can enhance migrants’ ability to navigate new contexts successfully [34]. These findings point to the strengths and resilience that migrant CYP can bring to their new environments [35], as well as the diverse and heterogeneous nature of migration experiences. In some cases, migration may be associated with positive health outcomes, as access to new resources, healthcare models, and treatment options in middle- to high-income countries can be transformative for migrant CYP with chronic illnesses [36]. Ultimately, whether experiences are positive or negative depends on the extent to which a migrant’s needs are met.

Given these complexities, research focused on migrant CYP with chronic illnesses is crucial. International organisations, activists, researchers, and healthcare professionals have consistently advocated for policies and interventions that address the healthcare needs of migrants, particularly those seeking asylum or sanctuary [14, 37]. However, migration is a multifaceted process, encompassing voluntary or forced movements, temporary or permanent stays, and legal or undocumented statuses, each influencing the experiences and needs of migrant groups. Recognising these distinctions is vital for developing health responses that effectively meet the varied needs of migrant CYP with chronic illnesses.

Despite the urgency of this issue, there has been no systematic synthesis of available evidence on international migrant CYP with chronic illnesses. This scoping review aims to map and summarise the breadth of evidence regarding this population’s post-migration experiences, health outcomes, and stakeholder recommendations. The review focuses on global evidence, incorporating perspectives from migrant CYP with chronic illnesses, their families and caregivers, and healthcare professionals. By doing so, this review informs future research, policy, and practice to improve the health and well-being of this vulnerable population.

## Methods

This scoping review follows the JBI methodology [38] to allow for the widest possible variety of studies to be included. Such a comprehensive approach is useful for gaining a thorough overview of a particular field, identifying gaps and informing future research directions, policymaking, and service development. The review methodology encompasses defining inclusion criteria, searching for evidence, screening and selecting relevant studies, conducting data extraction and synthesis, and co-creation with knowledge users or partners. A distinctive aspect of our scoping review was the continuous collaboration with a Patient and Public Involvement Advisory Group composed of migrant young people in the UK living with sickle cell disease. This continuous engagement ensured that the review remained relevant and responsive to real-world needs, grounded in the lived experiences of those with personal experience of migration [39]. The review was registered with Open Science Framework [40] (https://osf.io/hqt2c/) and reported in line with the PRISMA-ScR guidelines [41] (see Additional File 1 for reporting checklist).

### Inclusion and exclusion criteria

The inclusion criteria were set using the mnemonic described by JBI for question formulation for scoping reviews: population, concept, and context [38].

- *Population*: Migrant CYP were defined as anyone 25 years old or younger born outside their country of residence and had a chronic illness. This included those who had migrated with a known chronic illness or were diagnosed post-migration. Migrants were conceptualised inclusively to encompass a wide range of (1) reasons for migration, including refugees, asylum seekers, reunified children, EU migrants, economic migrants, and educational and healthcare migrants; (2) circumstances of migration, e.g. accompanied or unaccompanied; and (3) immigration status, e.g. documented or undocumented (see Table 1). Chronic illness was defined in line with the WHO’s definition of chronic disease as a health problem that requires ongoing management over a period of years or decades and cannot currently be cured but can be controlled with the use of medication and/or other therapies [42].
- *Concept*: The main study concept was the transition of CYP to a new country and having a chronic illness. We included papers that reported on any aspect of the health status and experiences of migrant CYP with chronic illnesses and their families/carers, including illness experiences, school, work and family life, social relationships and connectedness, care access and experiences, and identity and belonging. We also included papers that reported empirical data on experiences, views, outcomes, recommendations, and interventions related to the above population.
- *Context*: The review focused on international migration (with no country or region limit) and on all health-related contexts — individual, family/home, school, work, relationships, hospital, clinic, local authority, migration/detention camps/centres, and community based.

Further details on the inclusion and exclusion criteria can be found in Table 1.

**Table 1:** Specific eligibility criteria

Concept	Inclusion criteria	Exclusion criteria
Population	1. CYP who have moved to another country and have a chronic illness, aged 25 years or younger. The upper limit is consistent with the WHO definitions of young people 2. Short-term (3–12 months) and long-term (> 12 months) residents 3. Inclusive of the following: • International migrant — an individual who has moved from one country to another, typically for at least 1 year. E.g. foreign workers, international students, and family members joining relatives • Refugee — an individual who is forced to flee their home countries due to persecution, war, or violence and who crosses an international border to find safety • Asylum seeker — an individual who has sought international protection and whose claims for refugee status have not yet been determined • Economic migrant — an individual who moves primarily to improve their economic situation • Environmental/ecological migrant: an individual forced to leave their homes due to sudden or gradual changes in their natural environment/habitat that adversely affect their lives or living conditions • Undocumented migrant — an individual who resides in a country without legal authorisation. E.g. entered without inspection at an official checkpoint, overstayed visas, expired legal status, and pending legalisation • Unaccompanied migrant — a minor (< 18 years) who migrates to another country without the presence of a parent or legal guardian	Papers including participants over 25 years or with a mean age > 25 years or no data presented separately for those aged ≤ 25 years • CYP with chronic illness born in their country of residence but have at least one foreign-born, parent, i.e. second-generation migrant CYP • CYP with learning disabilities • CYP with a mental health condition
Concept	Papers reporting data on health status, experiences, views, recommendations, outcomes, and interventions from CYP, parents/families, and/or care professionals	
Context	Transnational migration	In-country migration
Study design	• Primary research • Comparative studies • Case studies	• Reviews • Opinion pieces (commentaries) without empirical data • Epidemiological studies

*CYP* Children and young people

### Information sources and search strategy

An information specialist (E. P.) conducted systematic searches for published evidence in February 2024 across seven databases — MEDLINE, Embase, PsycINFO, Cochrane Library, CINAHL, Social Science Collection, and Web of Science. Searches were conceptualised around ‘migrant’, ‘children’, ‘young people’ and ‘chronic illness’, using free text terms. Subject headings (e.g. MeSH) were also included for databases which offer this functionality. An initial search strategy, developed for MEDLINE and refined following consultation with the review team, was used as a template for the other database searches (see Additional File 2 for the full search strategies applied across the different databases). Searches were limited to papers available in the English language. No publication date or geographical limits were applied. Reference lists of included studies and existing reviews were also scrutinised to include additional relevant studies.

### Study screening and selection

**Fig. 1:**
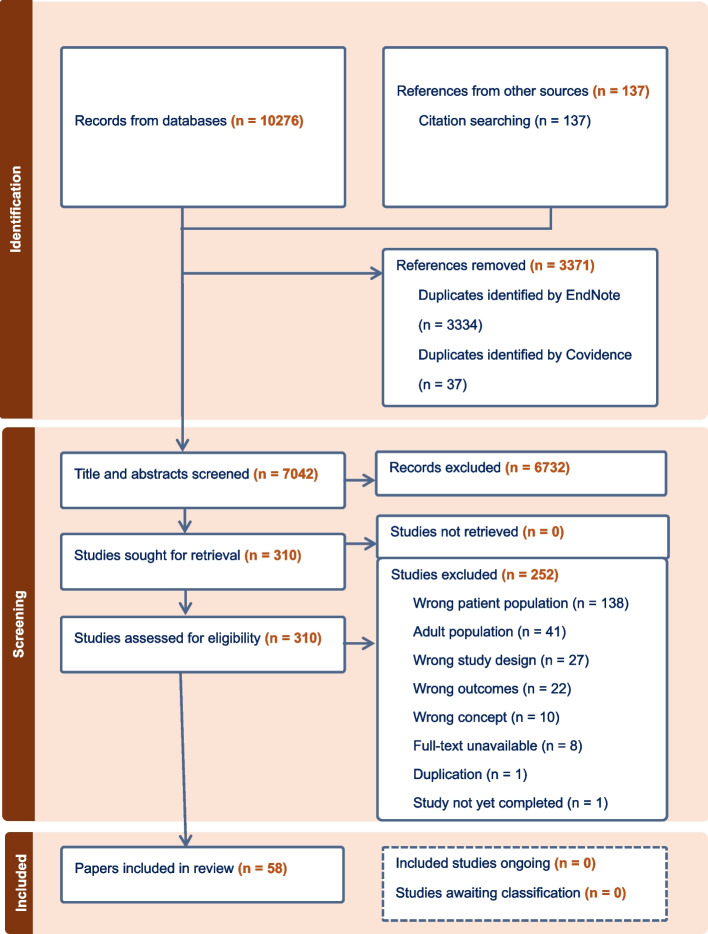
PRISMA flow diagram for the review. This diagram outlines the study selection process, including identification, screening, and inclusion stages

A total of 10,276 citation records were exported to EndNote X9®. After deduplication, 7042 records were exported to Covidence®, where titles and abstracts of the records were screened against the inclusion criteria. Full-text articles of 310 records that met the criteria were retrieved and screened against the inclusion criteria. Two reviewers (B. A. P. and L. H.) undertook study screening and selection independently. Each record was scrutinised for description and evidence that the paper was about first-generation migrant CYP with a chronic illness. In cases where this could not be determined explicitly or inferred, the paper was excluded. Any disagreements were discussed and resolved through consensus. The final number of included papers was 58 (from 57 studies). Refer to Fig. 1 for the PRISMA flow diagram.

### Data extraction

One reviewer (L. H.) primarily undertook extraction, charting, and summarising. An initial data extraction template was developed collaboratively by the whole team, focusing on study and participant characteristics and outcomes. The extraction form was piloted for further refinements and to ensure quality control and consistency. This entailed two reviewers (B. A. P. and L. H.) independently extracting data from 10% of the included papers and meeting afterwards to discuss any discrepancies. This ensured an unambiguous understanding of the meaning of each extraction domain and how differently reported data items should be categorised [43]. Data extraction and summary involved two steps:

1. Extracting and summarising key study characteristics, including methodology, methods, the study aims, study location, health system financing, conceptual model, participant characteristics, authors’ positionality and reflexivity, and the extent of the patient and public involvement (PPI) in the studies. The PROGRESS-Plus framework guided the extraction of participant characteristics to enable equity-focused analysis, ensuring sensitivity to diversity and inclusion within the evidence base [44]. Data extraction concerning the study authors’ positionality, reflexivity, and the presence and approach to PPI in the included studies was guided by the GRIPP2 reporting checklist [45]. As per scoping review guidance, formal quality appraisal was not undertaken.
2. Extracting and summarising data related to outcomes according to six main domains, with subdomains as follows: (1) clinical and health status (e.g. complications, quality of life, psychological outcomes); (2) healthcare utilisation (e.g. emergency visits, hospitalisation, barriers, facilitators); (3) healthcare access and experiences (e.g. treatment, communication, relationships, self-management, illness knowledge/meaning); (4) psychosocial experiences (e.g. stigma, identity and belonging, relationships, adjustment, school performance, social support); (5) care providers’ perspectives (challenges, barriers, facilitators — including those for interpreters); and (6) interventions/programmes (type, content, delivery)

### Analysis and synthesis of evidence

The second author (L. H.) followed the principles of narrative synthesis to analyse and draw together the evidence, as it permitted the synthesis of data generated via various methods. Specifically, Popay et al.’s [46] systematic approach was followed: involving preliminary analysis (extracting and tabulating the studies’ descriptive characteristics and producing a textual summary of results) and then exploring relationships (i.e. thematic analysis). The thematic findings were then mapped onto an adapted socio-ecological conceptual model described below, previous iterations of which have been well-refined by scholars in the fields of youth development and youth migration. This allowed for identifying key gaps in knowledge on the individual factors, actors, institutions, policies, and other influences which typically shape CYP’s development and life experiences.

### Conceptual framework

**Fig. 2:**
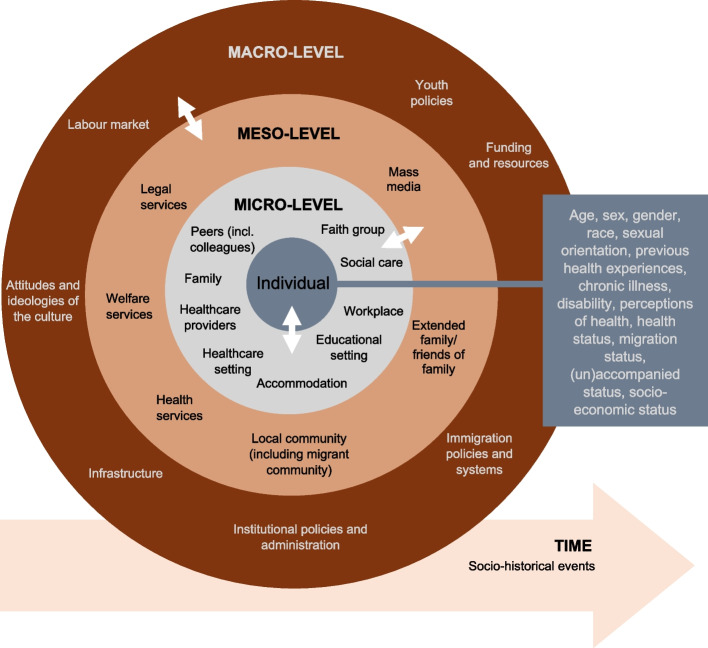
Socio-ecological conceptual model for the review. This model illustrates the multi-level factors influencing the post-migration experiences and health outcomes of children and young people with chronic illnesses

To analyse and synthesise the findings, this review employed an adapted version of Hunt et al.’s [47] socio-ecological framework (see Fig. 2). The framework builds on Bronfenbrenner’s [48] ecological systems theory, integrating elements from other relevant models [49–51]. The socio-ecological model is particularly effective for examining the complex interactions between individual, social, and structural factors that shape healthcare access and experience for migrant CYP with chronic illnesses. The framework identifies and maps out key factors and actors across different levels:

- *Individual level*: Personal characteristics of migrant CYP with chronic illnesses that influence their daily experiences and development
- *Micro level*: Interactions with close social actors like family, peers, and healthcare professionals (HCPs)
- *Meso level*: The role of broader community actors and institutions such as health, social, welfare, and legal services
- *Macro level*: Structural influences, including the host country’s policies, ideologies, and systems

The model emphasises the dynamic relationship between levels, recognising the tensions and supportive interactions among individuals, institutions, and structures. Furthermore, the framework accounts for socio-political events and personal histories, making it adaptable to diverse contexts. As such, using the model assists in highlighting gaps in knowledge, particularly at the individual level, where intersectional characteristics come into play. Similar models have been applied to refugee children’s psychosocial well-being [52] and immigrant adolescents’ transitions to adulthood [53]. For this study, we tailored Hunt et al.’s framework [47] to focus on healthcare rather than education. This involved additional individual-level characteristics such as chronic illness, health status, migration status, 'race', and socioeconomic status; changing ‘perception of education’ to ‘perception of health’ and ‘previous educational experience’ to ‘previous healthcare experiences’; shifting the health setting to the micro-level and replacing educators with HCPs, while including social care; expanding the meso level to include extended family; and incorporating ‘funding and resources’ and ‘infrastructure’ at the macro level.

While the framework proved useful, its application revealed certain challenges. Factors across different levels reported in the included papers often overlapped and intersected, making it difficult to clearly disentangle them. Consequently, the findings presented below reflected only the reported factors, conditions, outcomes, and their relationships within the included studies. The framework’s categorisation should, therefore, be viewed as a tool for organising data rather than a definitive method for disentangling complex interactions. It is also important to note that while the framework aids in clarification, real-world cases remain unique and require individualised approaches to examining and addressing complexities.

### Patient and public involvement

This review, part of a funded project titled *Young Migrants, Chronic Illness and Disability: The Case of Children and Young People with Sickle Cell Disease Who Migrate to the UK*, integrated a Patient and Public Involvement Advisory Group from the start. This board, comprising two young migrant men and two young migrant women with sickle cell disease (aged 16–22 years) recruited through two UK-based sickle cell-focused charities, was crucial in ensuring the review reflected lived experiences. They migrated from English-speaking African countries and had lived in the UK for between 2 and 15 years. Trained in the review process, they actively participated in setting the review objectives and data extraction and synthesis, highlighting overlooked areas such as psychological/mental health status and outcomes. Their insights directly informed the review’s outcomes and recommendations, ensuring relevance to the target population’s needs.

### Reflexivity and positionality

The review was conducted by a multi-professional (with experience in education and health and social care practice) and multi-disciplinary (chronic illness, disability, education, migration, evidence-based healthcare, CYP health research, healthcare communication/interactions) team of academic researchers, including an information scientist. Guided by the first author’s (B. A. P.) research focus on understanding and improving care experiences for CYP with chronic illnesses from marginalised backgrounds, the team’s diverse perspectives, expertise, and interests enriched the review process. Regular meetings, reviews, and feedback sessions allowed for continuous reflection on the review’s conduct, data analysis, and write-up, as well as how our individual and collective experiences and insights about the research area might influence the review, minimising undue influence and enhancing the credibility of the outcomes.

## Results

### Overview of the included studies

The 58 papers [54–111] (from 57 studies), all published in English between 1999 and 2023, predominantly focused on migration to or within high-income countries (*n* = 41), with the USA being the most represented country (*n* = 12). Studies in low-income settings primarily involved refugee-receiving countries like Türkiye, Jordan, and Lebanon. Over half referred to primary care contexts (*n* = 29), with cancer being the commonly reported chronic condition (*n* = 18). As detailed in Table 2, most studies (*n* = 39) focused on CYP, though their voices were rarely represented, with institutional records, inter(national) registries, or case studies being the main data sources. Less than half (*n* = 24) specified the migration status or reasons for migration. Instead, many used broad terms like ‘(im)migrants’ or proxies like country of birth or foreign language use to indicate immigration status (*n* = 30). Where specific categories of migrants were given, most studies referred to refugees or ‘displaced’ people (*n* = 19). Age was reported in 34 studies, with 25 focused on CYP aged 18 years or younger and 9 covering up to 25 years. Three studies covered the entire age range from 0 to 25 years.

**Table 2:** Overview of the included studies

Publication type	Journal article (*n* = 48) [54–101] Conference abstract (*n* = 8) [102–109] Chapter in an edited volume (*n* = 1) [110] Doctoral thesis (*n* = 1) [111]
Methodology	Quantitative (*n* = 33) [7–56, 56, 57, 57–60, 60–62, 62, 63, 63, 64, 64–66, 66, 67, 67–69, 69–76, 78, 79, 82–84, 88, 90, 91, 95, 97, 98, 100–105, 107–109] Qualitative (*n* = 22) [54, 58, 59, 61, 65, 68, 70, 72, 77, 80, 84–87, 89, 92–94, 101, 106, 110, 111] Mixed methods (*n* = 3) [55, 71, 96]
Purpose	To describe clinical cases (*n* = 21) [53, 54, 57, 64, 67, 70, 72, 73, 75, 77, 78, 85, 93–98, 101, 104, 105] To explore the effect of migration status (and country/region of origin) and other socioeconomic disparities on access and outcomes (*n* = 13) [59–61, 63, 66, 68, 76, 79, 80, 91, 99, 101, 102] To assess research or practice methods (*n* = 8) [58, 65, 71, 81, 87, 88, 92, 103] To investigate HCPs’ (including interpreters’) attitudes and challenges (*n* = 7) [52, 55, 62, 69, 74, 82, 83, 106] To describe and understand migrant CYP’s and their families’ perspectives, experiences, needs, access, and preferences (*n* = 4) [56, 84, 86, 89, 90, 107, 108]
Participant group	CYP (*n* = 39) [56, 57, 59, 60, 62–64, 66–68, 70, 73–76, 78, 79, 81–83, 87–91, 95, 97, 98, 100–105, 107–111] HCPs (*n* = 9) [54, 55, 58, 65, 72, 77, 85, 86, 94] Caregivers (*n* = 2) [61, 71] CYP & caregivers (*n* = 2) [69, 106] HCP & caregivers (*n* = 4) [84, 93, 96, 99] CYP, caregivers, & HCPs (*n* = 1) [92] HCP & community leaders (*n* = 1) [80]
Geographical focus	USA (*n* = 12) [55, 66–68, 70, 74, 77, 79, 80, 96, 97, 101] Sweden (*n* = 7) [65, 72, 73, 84–86, 89] Türkiye* (*n* = 7) [60, 75, 98–100, 104, 107] Jordan (*n* = 5) [56–58, 83, 90] UK (*n* = 4) [92, 95, 106, 111] Australia (*n* = 2) [59, 87] Canada (*n* = 2) [61, 71] Italy (*n* = 2) [62, 103] Lebanon (*n* = 2) [81, 91] Botswana (*n* = 1) [78] Chile (*n* = 1) [108] Denmark (*n* = 1) [63] Finland (*n* = 1) [76] France (*n* = 1) [69] Germany (*n* = 1) [102] Greece (*n* = 1) [109] Netherlands (*n* = 1) [94] New Zealand (*n* = 1) [93] Spain (*n* = 1) [82] Switzerland (*n* = 1) [54] Thailand (*n* = 1) [110] Multiple European countries (*n* = 3) [64, 88, 105] *Please note that while this paper uses the newly official name of Türkiye, many of the studies were conducted and published prior to this change — hence the use of ‘Turkey’ in some study titles and quotations
Chronic illness	Cancer (*n* = 18) [55, 58, 68, 69, 72, 75, 76, 82, 84–86, 90, 91, 94, 96, 98, 104, 107] HIV (*n* = 10) [64, 66, 67, 78, 87, 95, 97, 105, 110, 111] Asthma (*n* = 6) [62, 63, 65, 73, 93, 109] Type 1 diabetes (*n* = 5) [70, 83, 88, 102, 108] Various unspecified conditions (*n* = 5) [54, 74, 77, 80, 92] Sickle cell disease (*n* = 2) [62, 103] Heart disease (*n* = 4) [56, 57, 81, 106] Kidney disease/end-stage renal failure (*n* = 3) [60, 79, 101] Cystic fibrosis (*n* = 1) [100] Beta-thalassemia (*n* = 1) [99] Stroke and cerebrovascular disease (*n* = 1) [72] Chronic pain (*n* = 1) [59] Various severe blood disorders (*n* = 1) [59] Various long-term conditions (epilepsy, postoperative paraplegia, blue rubber bleb venous malformation, and cerebral paresis) (*n* = 1) [89]
Migration status of CYP	No specific status given (*n* = 34) [54, 55, 58, 61–66, 68–73, 76, 78, 80, 82, 84–86, 88, 92–96, 102, 105, 108–111] Refugee/displaced (*n* = 19) [56, 57, 59, 60, 67, 74, 75, 81, 83, 89–91, 98–100, 103, 104, 106, 107] International adoptee (*n* = 2) [67, 97] Undocumented (*n* = 2) [79, 101] Compact of free association migrant* (*n* = 1) [77] Visa holder (study, work, partner/family, permanent residency, tourist) (*n* = 1) [87] *A special condition for migrants from the Republic of the Marshall Islands and other Pacific Island sovereign states in the USA: ‘COFA [Compact of Free Association] migrants have the legal status of ‘lawfully present migrants’ rather than immigrants’ (McElfish et al., 2015, cited in Low et al., 2019, p. 54)

*CYP* children and young people, *HCP* Healthcare professional, *HIV* Human immunodeficiency virus, *UK* United Kingdom, *USA* United States of America

The most common study purpose was presenting clinical cases (*n* = 21), while only four studies aimed to describe migrant CYP’s and families’ experiences and needs. This, combined with the frequent use of medical records as data sources and using parents’ views as a proxy, revealed a substantial gap in the literature on CYP’s own perspectives (i.e. gathered via qualitative methods and from CYP directly). Additionally, using medical records as a data source raises ethical questions about patient consent for their use in research. Only three studies involved knowledge users or partners during the research process [80, 87, 92], typically limited to HCPs rather than CYP and/or caregivers. Their involvement was generally limited to consultation. Only four studies employed a conceptual or theoretical framework, which included bioethical principlism [77], the FOCUS consultation model [68], Goffman’s stigma [111], and AMOR II biopsychosocial protocol [82]. Only 10 studies [54, 58, 59, 65, 77, 80, 87, 93, 110, 111] discussed researcher identity and/or positionality, with nine noting efforts to address potential power, language, and disparities between researchers and participants. These highlight a need for more research that directly engages CYP and incorporates ethical considerations and theoretical frameworks to better understand and address the unique needs of migrant CYP with chronic illnesses.

As discussed in the following sections, most data were concentrated at the individual and micro levels of the socio-ecological model. See Table 3 below for a description of the results. At the individual level, 35 studies highlighted migrant CYP with chronic illnesses’ worse health status upon arrival and a higher rate of complications. Some studies (*n* = 10) discussed psychosocial challenges. However, the included studies rarely explored how individual-level factors such as age, gender, and ‘race’ specifically shaped the experiences of migrant CYP with chronic illnesses, revealing a significant gap in the literature. At the micro level, 42 studies focused on language and communication issues, cross-cultural (mis)understandings, healthcare costs, health insurance access, logistical and informational challenges, and limited understanding of new and complex systems. Some studies reported family dynamics and the role of parents in mediating care, but there was limited data on migrant CYP’s relationships with peers, schools, workplaces, or the broader community (*n* = 2). Faith groups and other association spaces (including online communities) were unexplored. At the meso level, eight studies explored the influence of extended family (whether together or abroad) and how a lack of coordination within and between institutions could disrupt migrant CYP’s care. However, the intersections of legal, health, and welfare services were underexplored. At the macro level, 12 studies mentioned infrastructural and resource challenges but only briefly addressed the socio-political contexts of host countries. The potential impact of hostile attitudes towards newcomers on their access to healthcare and services was notably underrepresented, indicating another significant gap in the existing evidence, considering the impact of such attitudes and ideologies.

**Table 3:** Results summary

Main themes	Subthemes
**Individual-level challenges and disparities**	*Poor health status on arrival*: Migrant CYP often arrived with worse health, including advanced disease stages and malnutrition, due to interrupted care or poor living conditions, though some fare comparably to children local to the new context
	*Delayed presentation, diagnosis, and referral*: Financial constraints, lack of awareness, and systemic barriers contribute to late diagnosis, advanced disease stages, and worse prognoses in migrant CYP with chronic illnesses
	*Disrupted treatment*: Language barriers, cultural factors, relocation, financial exclusion, complex healthcare systems, and stigma disrupt treatment plans, yet some migrant CYP benefit from high-quality care and improved outcomes in host countries
	*Differences in health outcomes*: Migrant CYP show varied health outcomes, with some experiencing higher mortality rates due to immigration status, socioeconomic factors, disrupted family structure, and adherence challenges and yet others achieving improved health post-migration due to increased access to medicines and medical technologies
	*Psychosocial challenges*: Migrants CYP with chronic illnesses often face psychosocial challenges, such as trauma, abuse, stigma, and inadequate mental health support, exacerbating their physical and emotional challenges
**Micro-level challenges**	*Language and communication*: Language barriers, including differences in languages and dialects, medical jargon, and varied communication practices/styles, compounded by limited access to interpreters and cultural mediators, often hindered effective communication between migrant CYP, their families, and healthcare providers
	*Cross-cultural (mis)understandings and social roles*: Cultural differences and gendered social roles led to misunderstandings, mistrust, and tension in transcultural care interactions between migrants, their families, and HCPs. Prejudices, mistrust, and racism were barriers to effective care, with both migrant families and HCPs at times reported to contribute to these challenges
	*Cost and health insurance*: Financial barriers, complex insurance processes, and unequal access to healthcare coverage significantly impact treatment continuity and outcomes for migrant CYP. Some countries or organisations provided financial support, but access was often limited to specific groups or conditions. HCPs played a key role in advocating for cost waivers and helping families access treatment through alternative means
	*Other access issues: information, bureaucracy, and physical mobility:* Navigating unfamiliar healthcare systems, administrative hurdles, and geographic inaccessibility of healthcare facilities limit timely and equitable care for migrant CYP
	*Family-related issues*: Family dynamics, financial stress, and cultural perceptions of illness influence how migrant CYP experience and manage their conditions
	*Relationships with peers, schools, workplace, and local community*: Stigma and stereotypes related to health conditions and ethnic identity challenged migrant CYP’s integration and well-being in social, educational, and workplace settings
**Meso-level challenges**	*Extended family influences*: Extended family members played a crucial role in supporting migrant CYP, offering financial, emotional, and logistical support for treatment and resettlement
	*Coordination within and between institutions*: A lack of coordination and information sharing between institutions (e.g. healthcare, resettlement, educational institutions) can lead to gaps in care, impacting the diagnosis, treatment, and overall well-being of migrant CYP with chronic illnesses. Good practices included having a contact point or referral service for medically complex migrant CYP with chronic illness to ensure efficient care transitions
**Macro-level challenges**	*Resources and infrastructure*: Financial and infrastructural limitations, especially in refugee-receiving countries, create significant barriers to providing adequate healthcare for migrant CYP, often relying on external funding sources. Already existing strains on healthcare systems in some host countries led to long waiting times for appointments, delaying necessary treatments and care
	*Socio-political context*: Political instabilities and civil unrest, as well as insufficient policies/commitments to integrate migrant health needs into host countries’ healthcare systems, impeded the development and sustainability of healthcare policies and support for migrants

*CYP* Children and young people *HCP* Healthcare professional

### Individual-level challenges and disparities

This first section discusses data from the studies on individual-level aspects — such as young migrants’ health status upon arrival and diagnosis — and how they interacted with factors such as their specific condition, migration background, and socioeconomic status.

#### Poor health status on arrival

Several studies reported that refugee and migrant CYP often arrived with worse health status than local children, including advanced disease stages, malnutrition, and severe complications. For example, some cancer patients were deemed inoperable or high risk, and those with HIV had lower odds of viral suppression [57, 78, 98, 99]. Many faced higher hospitalisation rates, recurrent illness episodes, early relapse, and increased infection rates due to disrupted care during migration or displacement, particularly within the first year, with younger and female migrants being more affected [62, 70, 76, 83, 99, 107, 109]. Long-term health issues and mental health challenges, such as behavioural conditions and educational delays, were also reported among some groups [88, 97]. Health disparities varied by country of origin and socioeconomic status, with migrant CYP from outside Europe in France, for example, less likely to have received prior cancer treatment [52, 69], while socioeconomic factors like partial funding and social welfare dependence contributed to worse health outcomes and higher hospitalisation rates [73, 78]. However, not all migrant CYP fared worse; some experienced mild medical issues that resolved over time, while others had health statuses comparable to local children [83, 98]. No clear patterns emerged from the literature to explain these variations.

#### Delayed presentation, diagnosis, and referral

Many studies reported delayed presentation, diagnosis, and referral for migrant CYP, leading to higher rates of complications and worse outcomes [64, 66, 100, 105]. Factors contributing to late presentation included financial constraints, lack of awareness about symptoms, missed screenings, challenges accessing free screening programmes, and low socioeconomic status [75, 79, 83]. Living in life-threatening conditions, with limited access to healthcare, transportation, and language barriers, further delayed care [98]. These delays, coupled with poor or missing medical records, often resulted in underdiagnosis or late diagnosis, worsening prognoses, and leaving many migrant CYP suffering or dying from severe outcomes such as organ failure or/being deemed inoperable [57, 60, 79].

#### Disrupted treatment

Several studies highlighted disruptions to treatment plans and loss of follow-up as significant issues for migrant CYP, often resulting in higher relapse rates [60, 95, 98, 104, 107, 109]. These disruptions were primarily due to language barriers, cultural differences, complex healthcare systems, and difficulty navigating services [98, 104]. Cultural beliefs and fear of stigma also influenced treatment decisions, with some families opting for traditional remedies or rejecting medical care altogether [60, 65, 92–94]. Practical barriers, such as relocation, immigration issues, and fear of job loss, contributed to delayed care and missed appointments [69, 75, 91, 98, 109]. Despite these challenges, many migrant CYP successfully accessed and adhered to treatment, often receiving care comparable to that of nonmigrants [60, 69, 75, 76, 96, 99, 103, 110]. In some cases, migrant CYP experienced shorter wait times and better outcomes, leading to the conclusion that care quality was not influenced by migration status in certain host countries [66, 78, 79, 90, 103, 110].

#### Differences in health outcomes

Migrant CYP showed varied health outcomes, with some reported to have higher (treatment-related) mortality rates due to the advanced disease stages and difficulties in following treatment plans [76, 98]. These were attributed to poor living conditions, limited access to healthcare and resources, communication barriers, and disrupted family structures [75]. However, outcomes varied within migrant groups. Older migrant CYP fared worse due to losing financial assistance [79], while migrants from outside Europe had poorer outcomes in Northern Europe than locals and other European migrants [64, 73], and adopted youth with HIV were reported to be significantly more likely to be virally suppressed than those not adopted due to adopted parents’ health insurance coverage [67]. However, some migrant CYP experienced improved health after migration, benefiting from better access to medicines, medical technologies, and enhanced self-management skills [67, 79, 99, 108] despite challenges with language, proper living conditions, and healthcare access [98]. These improvements led to comparable or better outcomes for some migrant CYP, with no clear patterns based on health status.

#### Psychosocial challenges

Beyond physical health, some studies also noted that migrant CYP with chronic illnesses face significant psychosocial challenges, including trauma, abuse, stigma, and inadequate mental health support, which compound their physical and emotional difficulties [58–60]. Refugees, for instance, often experience trauma from fleeing conflict, while other migrant groups, such as international adoptees, may have faced abuse or neglect [95]. Stigma, particularly for those with HIV, prevented seeking care, with some fearing deportation if their illness affects their visa status [54, 61, 87]. These psychosocial concerns were an even greater issue, given that mental health services and support may have been absent. This meant that for some, the impacts of trauma and adverse life events were not adequately addressed [92]. Many migrant youths also had to deal with wider life challenges, such as poor living conditions and difficulties in school [97, 104].

### Micro-level challenges and supports

This pertains to the interactions (or lack of them) migrant CYP have with people and institutions in their daily lives. This primarily refers to healthcare services, health personnel, family, and peers.

#### Language and communication

The most reported challenges and supports in migrant CYP’s and their families’ relationships and interactions with HCPs centred around language and communication. Issues included differences in languages and dialects, limited access to interpreters or cultural mediators, medical jargon, and varying communication styles [54, 60, 85, 99, 109]. Healthcare systems were reported to struggle to meet the linguistic needs of migrant families, compounded by cultural barriers, low health literacy, limited availability of local language training support, and discrimination, leading to discomfort, uncertainty, and a sense of lost control [65, 72, 80, 102]. Even when interpreters were available, challenges such as dialect differences, incomplete translations, and the balance between empathy and professionalism created further communication difficulties [58, 60, 65, 85]. The use of interpreters led to missed opportunities for building transcultural trust through information giving between migrants and HCPs [85]. In some cases, families preferred to use relatives instead of professional interpreters, raising concerns about privacy, confidentiality, the accuracy of translations, potential censorship, informed consent, adequacy of migrant CYP’s medical information and care situation, and issues with communicating outside approved frameworks [65, 77, 94]. These language challenges were exacerbated by organisational and time-related constraints, which meant that HCPs limited the detail they gave to migrant families [94] or expressed frustrations at how time-consuming their activities were with them [65]. To address these issues, some institutions provided multilingual printed materials, colour-coded pictorial treatment instructions, or scheduled social workers, while others tailored communication strategies to the child’s age and disease progression to improve emotional support, trust, ‘psychosocial adaptation’, and understanding of medical information [70, 82, 92, 106].

#### Cross-cultural (mis)understandings and social roles

Cultural differences were reported to play an important role in mediating interactions between families and HCPs, often leading to misunderstandings and challenges in providing effective care. These included mismatches between families’ care and treatment expectations and healthcare practices in host countries, such as families’ preferences for face-to-face doctor consultations or differing emotional expressions [58, 65, 72, 85]. Gendered roles in decision-making, especially within families, created further obstacles, with fathers sometimes taking dominant roles, even when mothers had more knowledge of the child’s illness [65, 84, 86]. Mistrust and prejudice, including racism, both from migrant families and HCPs, hindered effective communication and care [72, 85]. These issues were compounded by organisational constraints like time limitations and unfamiliarity with cultural beliefs, leading to stereotyping and frustration on both sides [54, 85]. However, good cross-cultural practices reported included the use of community advocates or interpreters as cultural mediators [72, 92], involving the extended family in care discussions [68], and fostering compassionate communication through verbal and non-verbal cues [68]. These approaches helped build trust and understanding, improving care delivery, especially when families wanted to discuss alternative treatments or other concerns [68].

#### Costs and health insurance (including public, private, and universal schemes)

Another major access issue reported was the cost of healthcare, coupled with the complexity of health and social insurance policies and processes. Financial barriers and complex insurance processes significantly affected treatment continuity and outcomes for migrant CYP. For instance, in Jordan, 12% of Syrian refugee children with heart disease died while waiting for health insurance coverage for surgery [57], while 20% of paediatric kidney transplant patients in the USA lost their grafts due to difficulties in affording immunosuppressive medications [79]. High healthcare costs, bureaucratic hurdles, and ineligibility for public or private insurance led to delays in treatment, lower retention in care, and poorer health outcomes, even in countries with universal healthcare systems [54, 59, 70, 78, 88, 92, 109]. Additional expenses, such as transportation and accommodation, further compounded access issues [78, 96]. Access to health insurance varied based on immigration status, nationality, age, and length of residence [79]. Refugees and asylum seekers were less likely to have coverage, and even when eligible, processing delays left them uninsured at critical moments [67, 70, 74, 77]. Some financial support schemes existed, including waived costs, free medicines, and subsidies from NGOs, governments, charitable organisations, or community support groups, but these were often restricted to specific groups or conditions [60, 87, 96, 98, 99, 101]. HCPs played a crucial role in advocating for migrant families, assisting with financial aid applications, finding alternative providers, and sometimes bending hospital rules to extend care or access resources [70, 77, 101, 107]. Their efforts helped mitigate financial barriers, ensuring that some migrant CYP received necessary treatment despite systemic limitations.

#### Other access issues: information, bureaucracy, and physical mobility

Many migrant CYP and their families were reported to struggle to navigate unfamiliar healthcare systems due to language barriers, limited health literacy, and a lack of culturally appropriate health information [54, 60, 70, 80, 89, 92]. Understanding insurance processes, appointment systems, and available support was particularly challenging [59]. Administrative hurdles, including incomplete or untranslated medical records, led to repeated procedures, delayed diagnoses, and treatment interruptions [54, 57, 60, 98]. Bureaucratic barriers, such as registration requirements and delays in obtaining parental consent, further postponed care [75, 98]. Geographic inaccessibility also limited timely treatment. HCPs willing to treat uninsured or undocumented migrants were often far from where families lived, while transportation costs and availability posed additional challenges [70, 78, 80, 96, 101]. These barriers collectively reduced access to specialist care and continuity of treatment, worsening health outcomes for migrant CYP [63, 78, 80, 83, 102].

#### Family-related issues: shame, finances, fear, and mediation

Family dynamics, financial constraints, and cultural perceptions of illness shaped how migrant CYP experienced and managed their conditions. While family networks provided essential support, they also created stress as relationships were renegotiated, families sought employment, and experienced prolonged separation from loved ones [61, 69, 82, 101]. Families played a key role in shaping CYP’s understanding of illness, sometimes reinforcing stigma or concealing diagnoses to protect them from distress [58, 72, 84, 93, 111]. Those with relatives abroad often withheld health information, limiting support networks [110]. Financial constraints were a major challenge, particularly for low-income and refugee families reliant on aid [82, 83, 93, 104, 107]. Caregivers sacrificed personal and professional opportunities to stay with their child in the hospital, increasing family stress [61]. Adjusting to a new healthcare system and multidisciplinary teams added to anxieties, especially for young refugee women prioritising family welfare over their own health [59, 62, 96]. Families also mediated interactions with HCPs, sometimes withholding information or modifying translations to maintain hope [72, 84]. While well-intended, these actions led to ethical dilemmas, confusion for migrant CYP, and tensions in care relationships [58, 93].

#### Relationships with peers, school, workplaces, and the local community

Outside their family, migrant CYP faced challenges with disclosure and managing their differences at work, school, and in the community due to stigma and stereotypes tied to their diagnosis and ethnic identity. These challenges were particularly pronounced for those who migrated at an older age [87, 92, 111]. At school, CYP with asthma, for instance, were reported to be often underestimated in physical activities, singled out, and overprotected in social settings [93]. Those with HIV feared disclosing their status to employers and worried about rejection and intolerance, even though secrecy was a way to exercise agency [111]. These experiences impacted the social and emotional well-being of the entire family [92].

### Meso-level challenges and supports

This addresses wider relationships and institutions around the migrant young person with chronic illness, such as those with extended family members and staff involved in, where relevant, their resettlement.

#### Extended family influences (whether together or abroad)

Extended family influenced both migrant CYP and their immediate families. Parents’ management strategies often shifted based on the strength and positivity of extended family influence [93]. Relatives, including spouses and grandparents, sometimes accompanied families for treatment, provided emotional support, raised funds, or offered local accommodation, easing financial strain on primary caregivers [96].

#### Coordination within and between institutions

A lack of coordination, continuity, efficiency, and organised efforts across institutions hindered the proper diagnosis and management of migrant CYP, leading to preventable morbidity and mortality [57, 74]. Gaps included poor information sharing in resettlement programmes, limited chronic disease screening and assessment of newcomers’ needs, and a shortage of case managers with health expertise. HCPs often lacked time to explore medically complex cases [74, 80]. Coordination issues also affected interpretation quality and created unclear responsibilities, such as school nurses feeling unable to administer medications [70]. A key good practice was a designated contact point or referral service as a ‘locus of expertise’ for medically complex migrant CYP to improve access, care transitions, communication, and triaging [74].

### Macro-level challenges

This focuses on how wider structural and political conditions were reported to influence migrant CYP’s access to and healthcare experiences.

#### Resources and infrastructure

At the macro level, studies highlighted the financial burden of providing care for newcomer CYP with chronic illnesses, particularly in major refugee-receiving countries like Türkiye, Jordan, and Lebanon [60, 75, 81]. Limited specialised hospitals and medical teams further strained healthcare systems [56, 57]. In Jordan, for instance, institutions relied on fragmented, nonofficial funding sources such as charities and UNHCR due to the absence of a centralised funding structure [56, 57]. Financial insecurities in host countries exacerbated these challenges. In Jordan and Lebanon, for example, limited national resources worsened access for migrant CYP with chronic illnesses [81, 83], while inadequate funding in Jordan restricted cancer treatment for displaced patients [90]. In Greece, economic instability directly impacted healthcare for migrant youth [109]. Even in high-income countries, publicly funded systems were under strain, leading to long wait times in the UK [92] and higher demand on US institutions treating undocumented youth due to the lack of or limited federal support [101].

#### Socio-political context

Beyond costs and infrastructure, the political climate and migration policies also shaped healthcare access. In Chile, migrant patients were only recently recognised as part of the public hospital system, complicating care coordination [108]. Uncertain immigration status delayed treatment initiation, particularly for asylum seekers awaiting decisions [88]. In Lebanon, broader civil disturbances further hindered the implementation of sustainable healthcare policies for all [88].

## Discussion

This review highlights the predominant focus in the literature on migrant CYP with chronic illnesses in high-income settings, often addressing individual and micro-level challenges. Studies commonly draw on institutional data or (inter)national registries to examine disparities in health status upon arrival, access to healthcare, adherence to treatment, and outcomes. Key barriers identified include language and communication [54, 77, 85, 93, 104] and coordination and cost [57, 70, 74, 96], but these challenges are interrelated, often compounded by immigration status, which impacts health insurance eligibility and coverage duration. Coordination issues can lead to higher costs for both host countries and families [57, 70, 74], and unfamiliarity with the host country’s healthcare system frequently results in overreliance on emergency care [54, 57, 60, 70, 98]. Migration status also correlates with socioeconomic status, with refugee CYP frequently living in overcrowded and unsanitary conditions that further hinder access to necessary care [75, 98].

The challenges faced by migrant CYP with chronic illnesses are exacerbated by their ongoing medical needs. While some identified issues, like healthcare access, long waiting times, and socio-political barriers [88, 88, 92], are common to all migrants, they take on particular urgency for migrant CYP. Delayed or interrupted care can worsen chronic conditions, potentially leading to serious complications or even preventable morbidity and mortality. Barriers such as limited access to specialist care, inadequate housing, and lack of culturally responsive healthcare can result in life-threatening situations. Additionally, the psychosocial burdens associated with managing a chronic illness in an unfamiliar environment can worsen physical health problems and hinder effective management.

The use of the socio-ecological model in this review made such interactions and influences visible at various social levels and allowed for gaps to be identified. For example, at the individual level, the influence of ‘race’ on migrant CYP with chronic illnesses’ experiences was rarely mentioned, neither was their country of birth or citizenship or, indeed, their own perceptions of health, illness, and appropriate treatment. At the micro level, there was very little data on the role of siblings, peers, faith groups and social workers, or migrant CYP with chronic illnesses experiences in educational or work settings. At the meso level, the studies omitted discussing the role of the local community in depth (including other migrants), legal and welfare services, and the impact of the media, for example. Then, at the macro level, very little work was found on state-level funding and resources, the influence of asylum and immigration policies (including definitions of citizenship), the accessibility of the labour market (in terms of employment rates and access and accommodation for workers with support needs), and cultural ideologies that informed a population’s response to migrants and refugees. From a methodological point of view, there was little qualitative enquiry focusing on migrant CYP’s own perspectives, little intersectional analysis (beyond age, sex, and gender), little clarity on terms used to describe migrants (such as ‘immigrant’ or ‘foreigner’), and little reflection on purpose, power relationships, and positionality.

Given the gaps identified, future research should adopt more comprehensive and rigorous approaches to examine the broader socio-political and economic contexts that influence the health of migrant CYP with chronic illnesses. This includes prioritising intersectional analyses that account for factors such as ‘race’, country of birth, sex, gender, and migrant CYP’s own perceptions of health and illness. Qualitative studies that capture the lived experiences of migrant CYP are essential to deepen understanding and highlight the unique challenges faced by these individuals. Engaging stakeholders, such as policymakers, healthcare practitioners, community support groups, and migrant families, in research design, conduct, and dissemination will ensure that studies address real-world needs.

The rising cost of treatments, especially for novel therapies like cancer care and gene therapies, presents an additional barrier to healthcare access for migrant CYP with chronic illnesses. These treatments are often unaffordable, particularly in low-income regions and refugee-receiving countries, where healthcare systems are already under strain. For many migrant families, the financial burden of treatment, coupled with the costs of migration itself, including travel, accommodation, and loss of income, makes seeking adequate care increasingly difficult. This financial strain emphasises the need for further exploration of universal health coverage models, particularly in host countries with limited resources, to ensure affordable and equitable healthcare access.

As migration continues to be a global reality, policymakers must recognise the complex and interconnected factors that influence the health outcomes of migrant CYP with chronic illnesses. Policies should address these populations’ intersecting challenges, ensuring equitable access to healthcare, improving coordination between health, social, immigration, and legal services, and addressing socioeconomic barriers such as housing and employment. Re-evaluating immigration and asylum policies is critical to mitigating their adverse effects on health, particularly in terms of access to care and health insurance coverage. Furthermore, policies must incorporate the voices and experiences of migrant CYP to ensure they are responsive to the needs of this vulnerable population.

Although the number of studies included in the review is high, the relevance of data on the specific experiences of migrant CYP with chronic illnesses was limited, with many studies covering a broad range of conditions and migration contexts, which means that findings may not be generalisable to all migrant CYP with chronic illnesses globally. Furthermore, excluding studies in languages other than English reduces the diversity of the evidence base. Despite these limitations, the review has notable strengths, such as the involvement of a Patient and Public Invovelment Advisory Group with migrant CYP living with sickle cell disease in the UK, its rigorous methodology, and its use of a framework to embed an equity and intersectional focus [112].

## Conclusions

This scoping review underscores the need for a broader, more inclusive approach to understanding the health challenges faced by migrant CYP with chronic illnesses. While significant progress has been made in identifying individual- and micro-level barriers, there is a critical need for future research to explore the meso- and macro-level factors influencing health outcomes, particularly in low-income regions which host the greatest number of migrants [113]. Intersectional analyses, qualitative methods that capture migrant CYP’s lived experiences, and collaborations with key knowledge partners and users will be essential in addressing the complex and interconnected challenges these young migrants face.

## Supplementary Information

Additional File 1. PRISMA-ScR Checklist

Additional File 2. Search methods for scoping review of migrant children/young people with chronic illness. Appendix – Database Search Strategies

## Data Availability

All data generated or analysed during this study are included in this published article and its supplementary files.

## Abbreviations

CYP: Children and young people
HCP: Healthcare professional
UNHCR: United Nations High Commissioner for Refugees
WHO: World Health Organization
