# Supplementary material for: Children and young people at the intersection of chronic illness and migration: a scoping review
Source: BMC Glob Public Health. 2025 Mar 3;3:14. doi: 10.1186/s44263-025-00131-3 (PMC11874703; doi:10.1186/s44263-025-00131-3)
Supplement: Supplementary file 2 — Additional File 2. Search methods for scoping review of migrant children/young people with chronic illness. Appendix – Database Search Strategies [file 44263_2025_131_MOESM2_ESM.docx]

**Search methods for scoping review of migrant children/young people with chronic illness**

Systematic searches for published evidence were conducted by an information specialist (EP) between 2^nd^ and 9^th^ February 2024.

Searches were conceptualised around the following facets, using free text terms including those below.

| Migrants | Children and young people | Chronic illnesses |
| --- | --- | --- |
| Migrant  Refugees  Immigrants  Asylum seekers | Children  Infants  Adolescents  Juvenile  Paediatric  Pediatric  Young people  Young person  Young adults | Chronic illness  Chronic disease  Chronic health conditions  *including…*  Asthma  Diabetes  Epilepsy  Rheumatic diseases  &  HIV |

Subject headings (e.g. MeSH) were also included for databases which offer this functionality.

An initial search strategy, developed for MEDLINE and refined following consultation with the project team, was used as the template for the other database searches. It was agreed that due to the high volume of studies about HIV, a targeted search was used for this condition, and the results were imported separately from the other illnesses.

Searches were limited to articles available in the English language. No publication date or geographical limits were applied. Results from each database search were exported to EndNote, and duplicates were identified and removed. Full search strategies are provided in the appendices / supplementary material.

| **Database name** | **Other chronic illnesses** | **HIV** | **TOTAL (all conditions)** |
| --- | --- | --- | --- |
| MEDLINE-ALL including In-Process and Epub Ahead Of Print (via OvidSP) | 2361 | 1217 | 3578 |
| EMBASE (via Ovid) | 1249 | 455 | 1704 |
| PsycINFO (via Ovid) | 485 | 343 | 828 |
| Cochrane Library (via Wiley) | 71 | 9 | 80 |
| CINAHL (via EBSCO) | 1029 | 541 | 1570 |
| ProQuest Social Science Collection (incl. ASSIA) | 738 | 721 | 1459 |
| Web of Science (Science Citation Index and Social Science Citation Index) | 858 | 199 | 1057 |
| **TOTAL (all databases)** | **6791** | **3485** | **10276** |
| **…after deduplication in EndNote** | **4633** | **2309** | **6942** |

**APPENDIX – DATABASE SEARCH STRATEGIES IN FULL**

**Ovid MEDLINE(R) ALL <1946 to February 01, 2024>**

1 Refugees/ 13569

2 exp "Emigrants and Immigrants"/ 15944

3 "Transients and Migrants"/ 14578

4 (migrant* or refugee* or asylum seek* or resettl* or post migration or host country or home country or destination country or new homeland or immigrant* or immigrat* or emigrat*).mp. 103554

5 1 or 2 or 3 or 4 103554

6 exp Chronic Disease/ 631984

7 exp Epilepsy/ 127973

8 exp Asthma/ 144440

9 Cystic Fibrosis/ 40491

10 exp Diabetes Mellitus/ 519748

11 Anemia, Sickle Cell/ 24483

12 Heart Defects, Congenital/ 60428

13 Thalassemia/ 11409

14 Inflammatory Bowel Diseases/ 32064

15 rheumatic diseases/ or arthritis, juvenile/ 36745

16 Dermatitis, Atopic/ or exp Eczema/ or exp Psoriasis/ 82725

17 6 or 7 or 8 or 9 or 10 or 11 or 12 or 13 or 14 or 15 or 16 1651788

18 (((chronic* or longterm or "long term") adj3 (disease* or condition* or ill*)) or chronic kidney failure or diabet* or epilepsy or sickle cell or congenital heart disease or thalass?emia or leuk?emi* or ((childhood or p?ediatric) adj2 (cancer* or neoplasm*)) or inflammatory bowel disease* or asthma* or cystic fibrosis or ((rheumatic or rheumatological) adj2 (disease* or disorder* or condition*)) or (juvenile adj3 (arthritis or oligoarthritis or polyarthritis or enthesitis or dermatomyositis or ankylosing spondylitis)) or JIA or septic arthritis or lupus or SLE or scleroderma or fibromyalgia or kawasaki or eczema or atopic dermatitis or psoria*).mp. 2701293

19 17 or 18 2930045

20 exp child/ or child, preschool/ or infant/ or adolescent/ 3672011

21 (teen* or adolescen* or child* or infant* or juvenile* or paediatric* or pediatric* or boy* or girl*).mp. 4757391

22 20 or 21 4757391

23 Young Adult/ or early adult*.mp. 1027814

24 (young adj3 (person* or people or adult*)).mp. 1138096

25 23 or 24 1143752

26 5 and 19 and 22 2332

27 5 and 19 and 25 956

28 limit 26 to english language 2103

29 limit 27 to english language 895

**30 28 or 29 2361**

31 exp *HIV/ or exp *hiv infection/ or (HIV or HIV1 or HIV2 or HIV+ or HIV-positive or AIDS or acquired immune deficiency syndrome or acquired immunodeficiency syndrome or acquired immuno-deficiency syndrome).ti. 358685

32 5 and (22 or 25) and 31 1351

33 32 not 30 1327

**34 limit 33 to english language 1217**

**Embase <1974 to 2024 Week 05> (run 5^th^ Feb 2024)**

1 exp migrant/ or exp forced migrant/ 50464

2 exp migration/ 51255

3 (migrant* or refugee* or asylum seek* or resettl* or post migration or host country or home country or destination country or new homeland or immigrant* or immigrat* or emigrat*).mp. 105299

4 1 or 2 or 3 129313

5 exp chronic disease/ or exp post-infectious syndrome/ 247810

6 exp epilepsy/ 277659

7 exp asthma/ 308349

8 cystic fibrosis/ 85925

9 exp diabetes mellitus/ 1241433

10 sickle cell/ 3429

11 exp congenital heart disease/ 199214

12 thalassemia/ 15088

13 exp inflammatory bowel disease/ 202983

14 rheumatic disease/ or exp juvenile rheumatoid arthritis/ 74298

15 exp atopic dermatitis/ or infantile eczema/ 58804

16 eczema/ 31643

17 exp psoriasis/ 114773

18 5 or 6 or 7 or 8 or 9 or 10 or 11 or 12 or 13 or 14 or 15 or 16 or 17 2688710

19 (((chronic* or longterm or "long term") adj3 (disease* or condition* or ill*)) or chronic kidney failure or diabet* or epilepsy or sickle cell or congenital heart disease or thalass?emia or leuk?emi* or ((childhood or p?ediatric) adj2 (cancer* or neoplasm*)) or inflammatory bowel disease* or asthma* or cystic fibrosis or ((rheumatic or rheumatological) adj2 (disease* or disorder* or condition*)) or (juvenile adj3 (arthritis or oligoarthritis or polyarthritis or enthesitis or dermatomyositis or ankylosing spondylitis)) or JIA or septic arthritis or lupus or SLE or scleroderma or fibromyalgia or kawasaki or eczema or atopic dermatitis or psoria*).mp. 4186618

20 18 or 19 4479194

21 juvenile/ or exp adolescent/ or exp child/ 4055851

22 (teen* or adolescen* or (child or children or childhood) or infant* or juvenile* or paediatric* or pediatric* or boy* or girl*).mp. 4724762

23 21 or 22 4997055

24 young adult/ 534537

25 (young adj3 (person* or people or adult*)).mp. 698449

26 24 or 25 698449

27 4 and 20 and 23 3456

28 4 and 20 and 26 642

29 limit 27 to english language 3206

30 limit 28 to english language 622

31 29 or 30 3472

32 exp *Human immunodeficiency virus/ 106977

33 exp *Human immunodeficiency virus infection/ 481924

34 exp *acquired immune deficiency syndrome/ 298557

35 (HIV or HIV1 or HIV2 or HIV+ or HIV-positive or AIDS or acquired immune deficiency syndrome or acquired immunodeficiency syndrome or acquired immuno-deficiency syndrome).ti. 345844

36 32 or 33 or 34 or 35 619761

37 23 or 26 5379092

38 4 and 36 and 37 2525

39 limit 38 to english language 2212

40 39 not 31 2105

**41 limit 31 to "remove medline records" 1249**

**42 limit 40 to "remove medline records" 455**

**---------------------------------------------------------------------------------------------------------------------------**

**APA PsycInfo <1806 to January Week 5 2024> *(searched 5^th^ Feb 2024)***

1 exp asylum seeking/ 1169

2 human migration/ or human displacement/ or exp immigration/ or refugees/ 42820

3 (migrant* or refugee* or asylum seek* or resettl* or "post migration" or "host country" or "home country" or "destination country" or "new homeland" or immigrant* or immigrat* or emigrat*).mp. 65430

4 1 or 2 or 3 67887

5 exp Chronic Illness/ 35324

6 exp Epilepsy/ 31514

7 exp Asthma/ 5346

8 cystic fibrosis/ 1015

9 exp Diabetes/ 21408

10 sickle cell disease/ 1285

11 exp Genetic Disorders/ 29871

12 exp Congenital Disorders/ 8913

13 ulcerative colitis/ 335

14 lupus/ 900

15 eczema/ 186

16 5 or 6 or 7 or 8 or 9 or 10 or 11 or 12 or 13 or 14 or 15 131455

17 (((chronic* or longterm or "long term") adj3 (disease* or condition* or ill*)) or chronic kidney failure or diabet* or epilepsy or sickle cell or congenital heart disease or thalass?emia or leuk?emi* or ((childhood or p?ediatric) adj2 (cancer* or neoplasm*)) or inflammatory bowel disease* or asthma* or cystic fibrosis or ((rheumatic or rheumatological) adj2 (disease* or disorder* or condition*)) or (juvenile adj3 (arthritis or oligoarthritis or polyarthritis or enthesitis or dermatomyositis or ankylosing spondylitis)) or JIA or septic arthritis or lupus or SLE or scleroderma or fibromyalgia or kawasaki or eczema or atopic dermatitis or psoria*).mp. 173438

18 16 or 17 222378

19 child health/ 1432

20 adolescent health/ 4062

21 early adolescence/ 3136

22 late adolescence/ 478

23 19 or 20 or 21 or 22 8750

24 (teen* or adolescen* or (child or children or childhood) or infant* or juvenile* or paediatric* or pediatric* or boy* or girl*).mp. 1283106

25 23 or 24 1283106

26 emerging adulthood/ 8347

27 ((young adj3 (person* or people or adult*)) or emerging adult*).mp. 262080

28 26 or 27 262080

29 4 and 18 and 25 462

30 4 and 18 and 28 131

31 limit 29 to english language 452

32 limit 30 to english language 129

**33 31 or 32 485**

34 exp *HIV/ 42525

35 (HIV or HIV1 or HIV2 or HIV+ or HIV-positive or AIDS or acquired immune deficiency syndrome or acquired immunodeficiency syndrome or acquired immuno-deficiency syndrome).ti. 45838

36 34 or 35 52148

37 4 and (25 or 28) and 36 347

38 37 not 33 344

**39 limit 38 to english language 343**

**The Cochrane Library (searched via Wiley, 4^th^ February 2024)**

ID Search Hits

#1 MeSH descriptor: [Refugees] explode all trees 281

#2 MeSH descriptor: [Emigrants and Immigrants] explode all trees 293

#3 MeSH descriptor: [Transients and Migrants] explode all trees 126

#4 ((migrant* or refugee* or (asylum next seeker*) or resettl* or "post migration" or "host country" or "home country" or "destination country" or "new homeland" or immigrant* or immigrat* or emigrat*)):ti,ab,kw (Word variations have been searched) 2326

#5 #1 or #2 or #3 or #4 2326

#6 MeSH descriptor: [Chronic Disease] explode all trees 43060

#7 MeSH descriptor: [Epilepsy] explode all trees 3456

#8 MeSH descriptor: [Asthma] explode all trees 15006

#9 MeSH descriptor: [Cystic Fibrosis] explode all trees 2361

#10 MeSH descriptor: [Diabetes Mellitus] explode all trees 45441

#11 MeSH descriptor: [Anemia, Sickle Cell] 4 tree(s) exploded 1058

#12 MeSH descriptor: [Heart Defects, Congenital] explode all trees 3183

#13 MeSH descriptor: [Thalassemia] 4 tree(s) exploded 536

#14 MeSH descriptor: [Inflammatory Bowel Diseases] explode all trees 4786

#15 MeSH descriptor: [Rheumatic Diseases] explode all trees 21755

#16 MeSH descriptor: [Arthritis, Juvenile] explode all trees 445

#17 MeSH descriptor: [Dermatitis, Atopic] explode all trees 2660

#18 MeSH descriptor: [Eczema] explode all trees 1458

#19 MeSH descriptor: [Psoriasis] explode all trees 4691

#20 ((chronic* or longterm or "long term") near/3 (disease* or condition* or ill*)):ti,ab,kw

OR

("chronic kidney failure" or diabet* or epilepsy or "sickle cell" or "congenital heart disease" or thalass* or leukemi* or leukaemi* or "inflammatory bowel disease" or asthma* or "cystic fibrosis" or JIA or "septic arthritis" or lupus or SLE or scleroderma or fibromyalgia or kawasaki or eczema or "atopic dermatitis" or psoria*):ti,ab,kw

OR

((childhood or pediatric or paediatric) near/2 (cancer* or neoplasm*)):ti,ab,kw

OR

(juvenile near/3 (arthritis or oligoarthritis or polyarthritis or enthesitis or dermatomyositis or "ankylosing spondylitis")):ti,ab,kw

OR

((rheumatic or rheumatological) near/2 (disease* or disorder* or condition*)):ti,ab,kw 292981

#21 #6 or #7 or #8 or #9 or #10 or #11 or #12 or #13 or #14 or #15 or #16 or #17 or #18 or #19 or #20 328286

#22 MeSH descriptor: [Child] explode all trees 81197

#23 MeSH descriptor: [Child, Preschool] explode all trees 39282

#24 MeSH descriptor: [Adolescent] explode all trees 136261

#25 ((teen* or adolescen* or child* or infant* or juvenile* or paediatric* or pediatric* or boy* or girl*)):ti,ab,kw (Word variations have been searched) 356259

#26 MeSH descriptor: [Young Adult] explode all trees 95088

#27 (young near/3 (person or people or adult*)):ti,ab,kw (Word variations have been searched) 119587

#28 ((early next adult*)):ti,ab,kw (Word variations have been searched) 329

#29 #22 or #23 or #24 or #25 or #26 or #27 or #28 418280

**#30 #5 and #21 and #29 71**

#31 MeSH descriptor: [HIV] explode all trees 4211

#32 MeSH descriptor: [HIV Infections] explode all trees 17667

#33 (HIV or HIV1 or HIV2 or HIV or "HIV positive" or AIDS or "acquired immune deficiency syndrome" or "acquired immunodeficiency syndrome" or "acquired immuno-deficiency syndrome"):ti,ab,kw (Word variations have been searched) 53194

#34 #31 or #32 or #33 53194

#35 #5 and #21 and #34 17

#36 #30 or #35 80

**#37 #35 not #30 9**

--------------------------------------------------------------------------------------------------------------------------

| **CINAHL via EBSCOHost (run 02/02/24)** | Friday, February 02, 2024 3:53:22 PM |
| --- | --- |

| **#** | **Query** | **Limiters/Expanders** |  | **Results** |
| --- | --- | --- | --- | --- |
| S1 | (MH "Refugees") | Expanders - Apply equivalent subjects Search modes - Boolean/Phrase |  | 9,379 |
| S2 | (MH "Immigrants+") |  |  | 18,549 |
| S3 | (MH "Emigration and Immigration") OR (MH "Transients and Migrants") OR (MH "Relocation") |  |  | 9,328 |
| S4 | migrant* OR refugee* OR asylum seek* OR resettl* OR "post migration" OR "host country" OR "home country" OR "destination country" OR "new homeland" OR immigrant* OR immigrat* OR emigrat* |  |  | 47,461 |
| S5 | S1 OR S2 OR S3 OR S4 |  |  | 49,100 |
| S6 | MH "Chronic Disease+" |  |  | 74,392 |
| S7 | MH "Epilepsy+" |  |  | 21,324 |
| S8 | (MH "Asthma+") |  |  | 37,768 |
| S9 | (MH "Cystic Fibrosis") |  |  | 8,628 |
| S10 | (MH "Diabetes Mellitus+") |  |  | 189,519 |
| S11 | (MH "Anemia, Sickle Cell") |  |  | 5,772 |
| S12 | (MH "Heart Defects, Congenital+") |  |  | 29,761 |
| S13 | (MH "Thalassemia") |  |  | 1,492 |
| S14 | (MH "Inflammatory Bowel Diseases+") |  |  | 19,617 |
| S15 | (MH "Rheumatic Diseases") OR (MH "Arthritis, Juvenile Rheumatoid") |  |  | 7,463 |
| S16 | (MH "Dermatitis, Atopic") OR (MH "Eczema") OR (MH "Psoriasis+") |  |  | 16,041 |
| S17 | S6 OR S7 OR S8 OR S9 OR S10 OR S11 OR S12 OR S13 OR S14 OR S15 OR S16 |  |  | 398,329 |
| S18 | ((chronic* OR longterm or "long term") n3 (disease* OR condition* OR ill*)) |  |  | 197,108 |
| S19 | (“chronic kidney failure” OR diabet* OR epilepsy OR "sickle cell" OR “congenital heart” OR thalass#emia) |  |  | 351,635 |
| S20 | (leuk#emi* or ((childhood or p#ediatric) n2 (cancer* or neoplasm*))) |  |  | 47,319 |
| S21 | (“inflammatory bowel disease*” or asthma* or “cystic fibrosis”) |  |  | 76,704 |
| S22 | (((rheumatic or rheumatological) n2 (disease* or disorder* or condition*))) |  |  | 9,294 |
| S23 | ((juvenile n3 (arthritis or oligoarthritis or polyarthritis or enthesitis or dermatomyositis or ankylosing spondylitis))) |  |  | 5,018 |
| S24 | (JIA or septic arthritis or lupus or SLE or scleroderma or fibromyalgia) |  |  | 56,575 |
| S25 | (kawasaki or eczema or atopic dermatitis or psoria*) |  |  | 28,370 |
| S26 | S18 OR S19 OR S20 OR S21 OR S22 OR S23 OR S24 OR S25 |  |  | 705,413 |
| S27 | S17 OR S26 |  |  | 739,045 |
| S28 | (MH "Child+") OR (MH "Infant+") |  |  | 761,604 |
| S29 | (MH "Adolescence+") |  |  | 610,253 |
| S30 | (teen* or adolescen* or (child or children or childhood) or infant* or juvenile* or paediatric* or pediatric* or boy* or girl*) |  |  | 1,472,454 |
| S31 | S28 OR S29 OR S30 |  |  | 1,472,454 |
| S32 | (MH "Young Adult") |  |  | 293,265 |
| S33 | (young n3 (person* or people or adult*)) |  |  | 344,253 |
| S34 | S32 OR S33 |  |  | 344,253 |
| S35 | S31 OR S34 |  |  | 1,608,195 |
| S36 | S5 AND S27 AND S35 |  |  | 1,040 |
| **S37** | **S5 AND S27 AND S35** | **Limiters - English Language** |  | **1,029** |
| S38 | S5 AND S27 AND S35 |  |  | 1,005 |
| S39 | (MM "Human Immunodeficiency Virus+") |  |  | 7,346 |
| S40 | (MM "HIV Infections+") |  |  | 76,360 |
| S41 | TI (HIV or HIV1 or HIV2 or HIV+ or HIV-positive or AIDS or "acquired immune deficiency syndrome" or "acquired immunodeficiency syndrome" or "acquired immuno-deficiency syndrome") |  |  | 94,722 |
| S42 | S39 OR S40 OR S41 |  |  | 111,440 |
| S43 | S5 AND S35 AND S42 |  |  | 543 |
| **S44** | **S5 AND S35 AND S42** | **Limiters - English Language** |  | **541** |
| S45 | S5 AND S35 AND S42 |  |  | 517 |

---------------------------------------------------------------------------------------------------------------------------

**Search Strategy PROQUEST Social Science Collection (run 04/02/24)**

| Set# | Searched for | Databases | Results |
| --- | --- | --- | --- |
| S5 | ((noft(refugee* OR migrant* OR immigra* OR emigra*) OR noft((asylum NEAR/3 seek*)) OR noft(resettl* OR "post migration" OR "host country" OR "home country" OR "country of origin" OR "destination country" OR "new homeland")) AND (noft((chronic* OR longterm OR "long term") NEAR/3 (disease* OR condition* OR ill*)) OR noft((childhood OR pediatric OR paediatric) NEAR/2 (cancer* OR neoplasm*)) OR noft(juvenile NEAR/3 (arthritis OR oligoarthritis OR polyarthritis OR enthesitis OR dermatomyositis OR "ankylosing spondylitis")) OR noft((rheumatic OR rheumatological) NEAR/2 (disease* OR disorder* OR condition*)) OR noft("chronic kidney failure" OR diabet* OR epilepsy OR "sickle cell" OR "congenital heart" OR thalass* OR leukemi* OR leukaemi* OR "inflammatory bowel disease" OR asthma* OR "cystic fibrosis" OR JIA OR "septic arthritis" OR lupus OR SLE OR scleroderma OR fibromyalgia OR kawasaki OR eczema OR "atopic dermatitis" OR psoria*)) AND ((noft(young NEAR/3 (person OR people OR adult)) OR noft(early adult*)) OR noft(teen* OR adolescen* OR child OR children OR childhood OR infant* OR juvenile* OR paediatric* OR pediatric* OR boy* OR girl*))) AND la.exact("ENG") | Education Collection, International Bibliography of the Social Sciences (IBSS), Politics Collection, Social Science Database, Sociology Collection | **738** |
| S14 | ((((noft(refugee* OR migrant* OR immigra* OR emigra*) OR noft((asylum NEAR/3 seek*)) OR noft(resettl* OR "post migration" OR "host country" OR "home country" OR "country of origin" OR "destination country" OR "new homeland")) AND (title(HIV OR HIV1 OR HIV2 OR HIV OR "HIV positive" OR "AIDS" OR "acquired immune deficiency syndrome" OR "acquired immunodeficiency syndrome" OR "acquired immuno-deficiency syndrome") OR mainsubject(HIV OR HIV1 OR HIV2 OR HIV OR "HIV positive" OR "AIDS" OR "acquired immune deficiency syndrome" OR "acquired immunodeficiency syndrome" OR "acquired immuno-deficiency syndrome")) AND ((noft(young NEAR/3 (person OR people OR adult)) OR noft(early adult*)) OR noft(teen* OR adolescen* OR child OR children OR childhood OR infant* OR juvenile* OR paediatric* OR pediatric* OR boy* OR girl*))) NOT ((noft(refugee* OR migrant* OR immigra* OR emigra*) OR noft((asylum NEAR/3 seek*)) OR noft(resettl* OR "post migration" OR "host country" OR "home country" OR "country of origin" OR "destination country" OR "new homeland")) AND (noft((chronic* OR longterm OR "long term") NEAR/3 (disease* OR condition* OR ill*)) OR noft((childhood OR pediatric OR paediatric) NEAR/2 (cancer* OR neoplasm*)) OR noft(juvenile NEAR/3 (arthritis OR oligoarthritis OR polyarthritis OR enthesitis OR dermatomyositis OR "ankylosing spondylitis")) OR noft((rheumatic OR rheumatological) NEAR/2 (disease* OR disorder* OR condition*)) OR noft("chronic kidney failure" OR diabet* OR epilepsy OR "sickle cell" OR "congenital heart" OR thalass* OR leukemi* OR leukaemi* OR "inflammatory bowel disease" OR asthma* OR "cystic fibrosis" OR JIA OR "septic arthritis" OR lupus OR SLE OR scleroderma OR fibromyalgia OR kawasaki OR eczema OR "atopic dermatitis" OR psoria*)) AND ((noft(young NEAR/3 (person OR people OR adult)) OR noft(early adult*)) OR noft(teen* OR adolescen* OR child OR children OR childhood OR infant* OR juvenile* OR paediatric* OR pediatric* OR boy* OR girl*)))) AND la.exact("ENG")) NOT stype.exact("Wire Feeds" OR "Encyclopedias & Reference Works" OR "Blogs, Podcasts, & Websites" OR "Speeches & Presentations") | Education Collection, International Bibliography of the Social Sciences (IBSS), Politics Collection, Social Science Database, Sociology Collection | **721** |

**WEB OF SCIENCE (run 09/02/24)**

# Web of Science Search Strategy (v0.1)

# Database: Web of Science Core Collection

# Searches:

1: (TS=(refugee* or emigrat* or immigrat* or emigrant* or immigrant* or "asylum seek*")) OR TS=(resettl* OR "post migration" OR "host country" OR "home country" OR "destination country" OR "new homeland" ) Date Run: Fri Feb 09 2024 19:21:57 GMT+0000 (Greenwich Mean Time) Results: 215726

2: TS=("chronic disease*" or "chronic condition*" or "chronic illness*" or "chronically ill" or "long term illness*" or "long term condition*" or diabet* or epilepsy or "sickle cell" or "congenital heart disease" or thalassemia or thalassaemia OR leukemia or leukaemia or "childhood cancer*" or "childhood neoplasm*" or "pediatric cancer*" or "paediatric cancer*" or "pediatric neoplasm*" or "paediatric neoplasm*" OR "inflammatory bowel disease*" or asthma* or "cystic fibrosis" OR "rheumatic disease*" or "rheumatic disorder*" or "rheumatic condition*" or "rheumatological disease*" or "rheumatological condition*" OR "juvenile arthritis" or "juvenile oligoarthritis" or "juvenile polyarthritis" or "juvenile enthesitis" or "juvenile dermatomyositis" or "juvenile ankylosing spondylitis" or "septic arthritis" OR lupus or scleroderma or fibromyalgia or eczema or "atopic dermatitis" or psoria* ) Date Run: Fri Feb 09 2024 19:24:21 GMT+0000 (Greenwich Mean Time) Results: 2519031

3: TS=(child or childhood or children or boy* or girl* or teen* or adolescen* or "young adult*" or "young people" or "young person*" or juvenile* or paediatric* or pediatric*) Date Run: Fri Feb 09 2024 19:25:24 GMT+0000 (Greenwich Mean Time) Results: 3314305

4: TI=(HIV or HIV1 or HIV2 or HIV+ or AIDS) Date Run: Fri Feb 09 2024 19:26:19 GMT+0000 (Greenwich Mean Time) Results: 423439

5: #3 AND #2 AND #1 Date Run: Fri Feb 09 2024 19:26:37 GMT+0000 (Greenwich Mean Time) Results: 1050

6: (#1 and #3 and #4) not #5 Date Run: Fri Feb 09 2024 19:27:19 GMT+0000 (Greenwich Mean Time) Results: 268

7: #3 AND #2 AND #1 and English (Languages) Date Run: Fri Feb 09 2024 19:27:38 GMT+0000 (Greenwich Mean Time) Results: 1004

8: #3 AND #2 AND #1 and English (Languages) and Editorial Material or Proceeding Paper or Early Access or Letter or Book Chapters or Note or Correction, Addition or Meeting Abstract (Exclude – Document Types) Date Run: Fri Feb 09 2024 19:27:59 GMT+0000 (Greenwich Mean Time) Results: 913

9: #3 AND #2 AND #1 and English (Languages) and Editorial Material or Proceeding Paper or Early Access or Letter or Book Chapters or Note or Correction, Addition or Meeting Abstract (Exclude – Document Types) and Emerging Sources Citation Index (ESCI) or Arts & Humanities Citation Index (A&HCI) (Exclude – Web of Science Index) Date Run: Fri Feb 09 2024 19:28:30 GMT+0000 (Greenwich Mean Time) Results: **858**

10: (#1 and #3 and #4) not #5 and English (Languages) Date Run: Fri Feb 09 2024 19:30:40 GMT+0000 (Greenwich Mean Time) Results: 249

11: (#1 and #3 and #4) not #5 and English (Languages) and Emerging Sources Citation Index (ESCI) or Conference Proceedings Citation Index – Science (CPCI-S) or Arts & Humanities Citation Index (A&HCI) (Exclude – Web of Science Index) Date Run: Fri Feb 09 2024 19:30:58 GMT+0000 (Greenwich Mean Time) Results: 215

12: (#1 and #3 and #4) not #5 and English (Languages) and Emerging Sources Citation Index (ESCI) or Conference Proceedings Citation Index – Science (CPCI-S) or Arts & Humanities Citation Index (A&HCI) (Exclude – Web of Science Index) and Book Chapters or Letter or Meeting Abstract or Editorial Material or Note (Exclude – Document Types) Date Run: Fri Feb 09 2024 19:31:29 GMT+0000 (Greenwich Mean Time) Results: **199**
